# Special Issue: Advancement of Functionalized Mineral Materials and Rock

**DOI:** 10.3390/ma16093375

**Published:** 2023-04-26

**Authors:** Xi Du, Youliang Chen, Tomás Manuel Fernández-Steeger

**Affiliations:** 1 Department of Civil Engineering, University of Shanghai for Science and Technology, Shanghai 200093, China; duxijl@163.com; 2 Institut für Angewandte Geowissenschaften, Technische Universität Berlin, 10587 Berlin, Germany

## 1. Introduction

Mineral materials have historically been extensively utilised in human society, and they hold significant prominence in various domains such as military, aerospace, electronics, and environmental conservation. The functionalisation theme has consistently underpinned the advancement of mineral materials. The functionalisation of mineral materials is a prominent and prospective trend that aligns with the objective of mineral material processing technology. Functionalizing mineral materials is crucial for meeting application needs and exploring new markets or applications in response to evolving industrial and societal demands. Therefore, it is crucial to take proactive measures in utilising effective mineral substances for energy conservation, fire prevention, filling and coating, environmental protection, energy storage, heat preservation, and other related applications [1,2,3,4,5].

This Special Issue covers numerous aspects of functional mineral research. These topics focus on various themes, including the analysis of mineralogical composition and its effects on the physico-mechanical properties of geomaterials, the investigation of the impact of triaxial stress on the damage creep behaviour of geomaterials, and the evaluation of the thermophysical and mechanical characteristics of rocks under high-temperature circumstances [6,7,8,9,10,11,12,13,14]. The research conducted on these topics also involves various experimental and numerical techniques, such as digital image processing, Discrete Element Method (DEM), and analytical solution and simulation [13,15,16,17,18]. Moreover, several research studies have explored the feasibility of utilising organic waste materials, including red mud, bauxite tailings, and zeolite, as prospective building materials or sorbents to eliminate petroleum substances from paved surfaces [8,14,19].

The articles featured in this Special Issue provide noteworthy insights into the understanding of how rocks and mineral materials respond to various environmental loading conditions. They underscore the significance of comprehending the physical and mechanical characteristics of these materials for the development and implementation of infrastructure systems. Approximately thirty international universities and scientific institutions have conducted research on the topic, demonstrating its popularity.

## 2. Short Description of the Articles Presented in This Special Issue

The issues of the original research papers published in this Special Issue of Materials, and deriving from almost 30 international universities and scientific centres, can be divided as follows:

- The physical and mechanical behaviour, fracture, damage, and evolution processes of geomaterials under various environmental and mechanical conditions, such as thermal, mechanical, chemical, and electrical environments [6,7,8,9,10,11,12,13,14,15,16,17,20,21].
- The reuse or recycling of waste materials in the field of civil engineering [8,14,19].
- Computational investigations of the behaviour of geomaterials under variable conditions [12,15,16,17,18,22].

Wang et al. [6] developed a numerical model utilising the Voronoi method in UDEC to represent the behaviour of rock under uniaxial compressive loading subsequent to heat treatment. The model was evaluated using empirical data and demonstrated satisfactory concurrence. A proposed criterion was modified to distinguish between cracks caused by shear and those caused by tensile forces. The research findings suggest that incorporating porosity in the modelling process is crucial. Furthermore, the Voronoi method is deemed to be more effective in generating precise outcomes. Additionally, the study reveals that thermal effects significantly impact the advancement of tensile contact, while the shear contact diminishes as the treatment temperature increases.

The coupling effect of thermal and stress damage in rock specimens after heat treatment is described by the TM (thermomechanical) unified constitutive model developed by Wang et al. [7]. As confirmed by empirical findings, the model effectively depicts the complete process, encompassing compaction through post-failure phases. The equation for total damage evolution can be applied to determine the correlation between the ratio of total damage evolution and the axial strain of granite under varying temperatures. Results show that the peak of the total damage evolution ratio occurs later and decreases with increasing temperature.

The research conducted by Ou et al. [8] explores the mechanical characteristics of red mud and bauxite tailings mud with the aim of determining their suitability as subgrade materials in construction projects. This research investigates the mechanical characteristics of various materials and their suitability for implementation in subgrade functions. The findings indicate that the utilisation of red mud and bauxite tailings mud as subgrade materials can be a viable option, providing advantageous outcomes in terms of both ecology and finance for construction projects.

The study conducted by Ou et al. [9] investigates the engineering characteristics and microscopic mechanism of foam light soil that incorporates red mud and bauxite tailings, with the aim of exploring its potential use in geotechnical engineering applications. According to the research, the incorporation of red mud and bauxite tailings into foam lightweight soil resulted in enhancements in its mechanical characteristics, including, but not limited to, strength and stability. The utilisation of this substance in slope stabilisation and other geotechnical engineering applications is proposed by the authors. The enhanced characteristics were attributed to the establishment of a compact particle network and the occupation of voids by red mud and bauxite tailings, as evidenced by a microscopic examination.

Liu et al. [15] utilised digital image processing techniques on a grain block model to conduct an evolutionary investigation of the different microcracks that exist in granite. The researchers employed computed tomography (CT) scanning to acquire high-resolution imagery of the granite specimens, which were subsequently subjected to image processing techniques for the purpose of detecting and evaluating microcracks. The findings indicate that the microcracks displayed varying dimensions and orientations, and their progression was impacted by the granite’s heterogeneity. This research offers valuable insights into the mechanisms underlying the formation and evolution of microcracks in granite.

Shi et al. [10] conducted an investigation on the breaching process of landslip dams using physical flume tests. The study took into account the impact of different grain compositions and inflow conditions. The study’s findings indicate that the process of breaching was impacted by the interaction between water and sediment, and exhibited variability based on the type of dam material and the rate of inflow discharge. The study has identified three distinct stages of the breaching process. Furthermore, it has been observed that the final breach of dams with a higher fine content exhibited a deeper and narrower morphology. The erosion resistance of the materials used in dams was observed to increase in the direction of flow, while the lateral collapse of the dam was found to be influenced by the type of material used.

The impact of the mineralogical composition of commercial ornamental granite rocks on their physical and mechanical properties was investigated by Alzahrani et al. [11]. The influence of certain minerals, namely quartz, feldspar, and biotite, significantly impacts various characteristics such as the thermal expansion coefficient, spectral reflectance, hardness, compressive strength, and flexural strength. The aforementioned discoveries have the potential to assist in the process of choosing and categorising ornamental granite stones for construction.

The present study, carried out by Rao et al. [12], endeavours to examine the process of rock failure when subjected to high-voltage electropulse through the utilisation of analytical techniques and simulations. The proposed approach integrates the RLC equivalent circuit, a shock wave model, and rock failure characteristics to describe the damage to rock induced by electropulse. A numerical model was built using granite as an example to examine the growth process of cracks when subjected to an electric pulse. The proposed methodologies can accurately calculate the progressive failure process of granite and reveal the mechanism of rock failure under electropulse.

The study conducted by Wu et al. [16] provides a semi-analytical approach to determine the shock wave pressure and radius of the plastic zone in soil resulting from lightning strikes. The proposed solution considers the soil as a homogeneous and isotropic medium and takes into account the effect of the lightning current waveform, soil properties, and the distance between the lightning channel and the ground surface. The results are compared with existing experimental data and show a good agreement, demonstrating the effectiveness of the proposed method.

Under constant vertical stress, Wang et al. [13] conducted suction-controlled swelling deformation experiments on compacted bentonite specimens. Four typical void ratio–suction curve morphologies were observed, each revealing distinct swelling–collapse behaviour based on the suction and OCR (over-consolidation ratio). Various forms of hydration deformation curves were effectively simulated by defining a swelling index and developing equations for characterising swelling–collapse characteristics.

Li et al. [22] investigated the mechanical behaviour of talus-like granite formations, which are pervasive in southern China and have caused significant engineering catastrophes. Experimental experiments and numerical modelling were utilised to investigate the shearing characteristics of talus-like rock masses. In contrast to soil–rock mixtures, the results demonstrated that the strength parameters were proportional to the block content. Moreover, the research demonstrates a clear relationship between internal structure, fabric stability, and block content during shearing.

Chen et al. [17] conducted a quantitative analysis of micro and macro mechanical parameters using the PFC^3D^ flat-joint model. The aforementioned model was employed to simulate the mechanical response of a granular substance subjected to diverse loading conditions. The findings indicate that the model effectively forecasted the macroscopic mechanical characteristics of the material and the microstructural change that occurred during the deformation process. This research offers valuable insights into the behaviour of granular materials, which can be utilised in the development of advanced materials for various engineering applications.

Without the energy-intensive calcination procedure, Pabiś-Mazgaj et al. [14] demonstrated that creating an effective zeolite-based granular petroleum sorbent from waste dust is possible. The team utilised pressure agglomeration to create sorbent agglomerates that met the minimum weight requirement of 50% for oil absorbency efficacy. According to the research, the optimal combination for agglomeration technology consisted of 6% C-binder and 20–20.6% hydration, yielding comparable surface areas to commercial sorbents. Significant potential exists for the eco-friendly solution to reduce greenhouse gas emissions and refuse material.

The research conducted by Zhang et al. [18] provides a comprehensive analysis of the characteristics of sandy soil in relation to the excavation of a circular tunnel. Using simulations of 2D discrete element modelling, the effect of different particle shapes, particularly the aspect ratio and convexity, on the ground reaction was studied. The study examined the microscopic and macroscopic characteristics of sand, such as soil pressure fluctuations, void ratio, and particle rotation. The investigation determined that elongated particles cause asymmetric ground reactions and that a decreasing aspect ratio or increasing convexity increases ground settlement. In addition, it can be observed that particles with an elongated shape generate a greater degree of arching pressure on the lining of a tunnel compared to particles with a more spherical shape.

Chen et al. [20] proposed a damage creep constitutive model that considers the interaction between rock acid corrosion and actual triaxial stress and is in agreement with the experimental findings. The model has been demonstrated to effectively forecast the creep response of rocks subjected to varying stress states. The model exhibits potential applications in the realm of underground construction and in the analysis of geologic phenomena.

Dong et al. [21] conducted thermophysical and mechanical experiments on sandstone samples at temperatures ranging from room temperature to 1000 °C. The thermophysical properties of sandstone following a high-temperature treatment and the temperature-dependent changes in mechanical properties were analysed. The results indicate that as the temperature increases, the specific heat capacity and thermal expansion coefficient of sandstone samples subjected to a high-temperature treatment first increase and then diminish, while the thermal conductivity decreases gradually. The increase in temperature contributes to the transformation of sandstone’s failure mode from brittle to ductile.

Finally, Ivanova et al. [19] focused on recycling mining residues containing serpentine minerals and obtaining a granular magnesium silicate reagent for high-metal-content cleaning solutions. The study demonstrated the significance of material selection when manufacturing a granular magnesium silicate reagent. Chrysotile has been found to be a viable option for the manufacturing of granular materials, while Lizardite exhibits high activity and is recommended for utilisation in fractionated granules. Antigorite has the potential to be employed in the production of magnesium and silicate commodities.

## 3. Conclusions

The selection of the topic “Advancement of Functionalised Mineral Materials and Rock” as a Special Issue was deemed appropriate given the considerable volume of research articles that have been published on this subject matter. The advancement of functional mineral materials and rocks exhibits significant potential for sustainable development. Researchers can enhance their efficacy and introduce novel functionalities, rendering them suitable for diverse applications across various domains. It is expected that the potential applications of these materials will broaden with the progression of additional research. This will result in more significant innovation in fields such as building energy and environmental sustainability. In conclusion, the utilisation of functional mineral materials and rocks exhibits the potential to advance the growth of a sustainable and ecologically responsible global society.
